# Psychological support to relatives of critically ill patients with COVID-19

**DOI:** 10.31744/einstein_journal/2020CE6032

**Published:** 2020-08-28

**Authors:** Marcos Roberto Tovani-Palone, Sajjad Ali

**Affiliations:** 1 Faculdade de Medicina de Ribeirão Preto Universidade de São Paulo Ribeirão PretoSP Brazil Faculdade de Medicina de Ribeirão Preto, Universidade de São Paulo, Ribeirão Preto, SP, Brazil.; 2 Ziauddin University KarachiSindh Pakistan Ziauddin University, Karachi, Sindh, Pakistan.

Dear Editor,

Due to the pandemic of the coronavirus disease 2019 (COVID-19), thousands of scientific studies,^(1,2)^ as well as diverse treatment protocols,^(3,4)^ have been reported in the literature. So far, in the absence of vaccines against the severe acute respiratory syndrome coronavirus 2 (SARS-CoV-2), it has been a routine to care for severe COVID-19 patients at intensive care units, who are on invasive mechanical ventilation for several days; on average, during three weeks.^(1)^ This phase of the treatment will certainly imply on important psychologic impacts involving the relatives of these patients, since visits are not allowed, and there is no specific medication against SARS-CoV-2. Furthermore, inappropriate media coverage regarding the pandemic may exacerbate such impacts.^(5)^ Hence, although not very often addressed in the literature, it is worthy stressing the importance and need to provide psychological support to relatives of severe patients during hospitalization.
