# Supplementary material for: Psychological support to relatives of critically ill patients with COVID-19
Source: Einstein (Sao Paulo). 2020 Aug 28;18:eCE6032. doi: 10.31744/einstein_journal/2020CE6032 (PMC7453830; doi:10.31744/einstein_journal/2020CE6032)
Supplement: Translation

# Translation: Apoio psicológico a familiares de pacientes graves com COVID-19

**Authors:** Marcos Roberto Tovani-Palone, Sajjad Ali

**Affiliations:** 1 Brasil Faculdade de Medicina de Ribeirão Preto, Universidade de São Paulo, Ribeirão Preto, SP, Brasil.; 2 Paquistão Ziauddin University, Karachi, Sindh, Paquistão.

Caro Editor,

Diante da pandemia da doença pelo coronavírus 2019 (COVID-19), milhares de estudos científicos têm sido divulgados na literatura,^(1,2)^ bem como variados protocolos de tratamento.^(3,4)^ Até o momento, na ausência de vacinas contra o coronavírus da síndrome respiratória aguda grave 2 (SARS-CoV-2), é corriqueiro o atendimento a pacientes graves com COVID-19 em unidades de terapia intensiva com uso de ventilação mecânica invasiva por vários dias – em média durante 3 semanas.^(1)^ Essa fase do tratamento deve certamente implicar em importantes impactos psicológicos envolvendo os familiares desses pacientes, dado que visitas não são permitidas e ainda não há um medicamento específico contra o SARS-CoV-2. Em acréscimo a isso, coberturas inadequadas da mídia referentes à pandemia podem vir a gerar uma exacerbação desses impactos.^(5)^ Nesse sentido, embora ainda pouco discutidas na literatura, vale destacar a importância e a necessidade do oferecimento de suporte psicológico a familiares de pacientes graves durante o período de hospitalização nos serviços que estão atendendo esses casos.
